# Temporal dynamics of teen crisis help-seeking following hurricanes: A structural topic model analysis

**DOI:** 10.1371/journal.pdig.0001393

**Published:** 2026-05-12

**Authors:** Margaret M. Sugg, Sophia C. Ryan, Jennifer D. Runkle, Charlie S. Reed, LaVondra Dobbs, Victoria Schwandt

**Affiliations:** 1 Appalachian State University Department of Geography and Planning, Boone, North Carolina, United States of America; 2 University of North Carolina at Chapel Hill Department of Geography and Environment, Chapel Hill, North Carolina, United States of America; 3 North Carolina State University, North Carolina Institute for Climate Studies, Asheville, North Carolina, United States of America; 4 VIA LINK, Covington, Louisiana, United States of America; Xinjiang Medical University Affiliated First Hospital, CHINA

## Abstract

Extreme weather events pose significant mental health risks for youth, yet few studies have examined the temporal dynamics of crisis help-seeking following multiple hurricanes using real-time anonymized transcripts. We analyzed 2,149 anonymized crisis text conversations from Louisiana youth (2018–2023) using structural topic modeling (STM) to identify thematic patterns and temporal trajectories following six hurricanes, including Hurricane Ida. Conversations were assigned to the closest hurricane using a temporal proximity algorithm and classified into eight mutually exclusive recovery periods based on days elapsed since landfall, ranging from the immediate hurricane period (days −1 to +2) through beyond one year post-exposure (>365 days). Compound disaster effects (hurricane + COVID-19) were also examined. We identified 12 distinct crisis topics organized into four domains (Crisis, Coping, Stressor, Resource/Process) and documented a systematic “three waves” pattern of post-disaster psychological response. Wave 1 (Immediate: 0–30 days) was characterized by resource-seeking, with Crisis Hotline Protocol (17.4%) and Louisiana Services (9.8%) peaking during active storms. Wave 2 (Delayed: 31–90 days) revealed the emergence of acute crises, with Suicide Ideation & Self-Harm increasing from 5.1% during hurricanes to 8.9% at 61–90 days post-landfall (p = 0.006). Wave 3 (Long-term: 3 + months) was dominated by interpersonal stressors peaking at >12 months, including Relationships (17.8%) and Family Conflict (10.3%), with Abuse & Safety Concerns showing delayed emergence at 9 + months. Compound disasters (74% of sample) were associated with elevated Grief (+3.3 percentage points) and Abuse & Safety Concerns (+1.8 percentage points). Crisis text data reveal distinct temporal phases of youth mental health help-seeking following hurricanes, with a critical delayed suicide ideation peak at 61–90 days. These findings suggest potential windows for stepped-care frameworks and demonstrate the utility of crisis hotline data as a real-time tool for disaster mental health surveillance.

## Author summary

In this study, we analyzed over 2,000 anonymous crisis text conversations from Louisiana youth spanning six hurricanes between 2018 and 2023, including Hurricane Ida. Using a computational text analysis method, we identified 12 recurring themes in these conversations and tracked how they shifted across different stages of disaster recovery. Our findings suggest a consistent “three waves” pattern of psychological distress. Immediately after exposure, young people reach out primarily for practical resources and information. One to three months after exposure, conversations shift toward mental health crises, with suicidal thoughts peaking around 61–90 days after landfall. Over the longer term (e.g., 9 + months), relationships, family conflicts and abuse emerged. When hurricanes overlapped with the COVID-19 pandemic, youth showed higher rates of grief and abuse-related distress. These findings highlight specific windows of heightened vulnerability and suggest that crisis text lines could serve as a powerful early-warning system for disaster mental health response.

## Introduction

Extreme weather events are a growing public health concern in the US, with tropical cyclones among the most destructive to US coastlines, and are projected to intensify in the coming decades [1]. These events cause physical destruction and create significant psychological distress among affected populations [2–4]. The mental health impacts of such disasters include increased rates of anxiety, depression, and post-traumatic stress disorder [4–7], with evidence from numerous disasters, including tropical cyclones [6,8], winter weather [7], and wildfires [9,10].

Young people are particularly vulnerable to the mental health consequences of extreme weather [11,12]. This increased vulnerability stems from a combination of factors, including ongoing brain development that can affect emotional regulation and coping [13], limited autonomy and control over living situations [14,15], and a lack of experience in managing stress and adversity [16]. These developmental factors intersect with social determinants of health, such as socioeconomic status, race and ethnicity, and sexual and gender minority identity, to shape young people’s resilience or susceptibility to climate-related stressors [17].

Although the association between extreme weather and mental health is well established among youth and other subpopulations [12], relatively few studies have examined the mental health impacts of extreme weather events such as tropical cyclones using data sources beyond surveys [18], small-sample studies [4], or traditional hospital administrative datasets [6,18]. Existing evidence nonetheless indicates substantial mental health burdens among youth and young people following these events (e.g., [6]). However, much of this literature lacks a temporal perspective, limiting insight into post-disaster mental health trajectories, precipitating contexts, and critical windows for intervention in this high-risk population. This gap is notable given prior trajectory-based survey research demonstrating dynamic and heterogeneous post-traumatic stress responses following hurricane exposure [19–21].

Most recently, research has leveraged social media data (e.g., Twitter) to address this knowledge gap, capturing dynamic population-level impacts on affected communities [22,23] and offering unique, nuanced insights into outstanding post-disaster population health needs. Yet, additional research is needed that leverages diverse real-time datasets to identify correlates of post-disaster adolescent mental health that may inform decisions about the timing of interventions for individuals, families, and/or communities. Emerging data sources, such as anonymized text conversations from crisis hotlines, can provide essential insights into crisis events and help-seeking behaviors during extreme events [6,7,24–26]. In particular, anonymized crisis hotline data is uniquely poised to shed light on population-level help-seeking, including precipitating contexts, crisis concerns and needs, and lived experiences. Crisis hotline data have been leveraged to identify latent subgroups and topics [27,28], quantify how crisis concerns and help-seeking behaviors change in the context of an extreme weather event [6,7,24,25], and characterize indirect psychological effects of climate change [29]; yet to the authors knowledge these data have yet to be leveraged to capture dynamic population-level trends in help-seeking contexts.

More specifically, topic modelling approaches (e.g., latent topic modelling, structured topic modelling, dynamic topic modelling) have emerged as a methodology for understanding changes in mental health and behavioral crises across unique data sources such as social media and help-seeking datasets [30–32]. Topic modelling is a machine learning technique that uses text data to identify latent topics [30,33]. Structural topic modelling (STM) approaches take this a step further, incorporating document (or transcript) level variables to investigate how topics change over time and across populations (e.g., [31]). In the context of disaster mental health, leveraging these topic modelling approaches can provide novel insights into dynamic crisis responses, precipitating contexts, and critical intervention periods, contributing to persistent knowledge gaps.

This analysis aims to model the psychological impacts of exposure to tropical cyclones among youth using anonymized crisis hotline transcripts from a TeenTextLine, capturing dynamic transitions in crisis help-seeking contexts. Leveraging real-time crisis transcript data reveals nuanced insights into youth crises and how these change in the context of exposure to tropical cyclone events. Specifically, this research is guided by the following questions: How do mental health discussion topics differ across hurricane exposure periods? Do cumulative exposure time windows show a “dose-response” in topic prevalence or mental health severity? Results may identify critical intervention periods and have implications for targeted crisis resource allocation and community resilience during the multiple phases of disaster response and recovery.

## Data

### Help-seeking data

VIA LINK (VL) is a free, confidential, 24/7 crisis service organization based in Louisiana that provides resources, assistance, and emotional support to individuals in need, partnering with national hotlines 988 Lifeline - the National Suicide and Crisis Lifeline (www.vialink.org), and delivering targeted services, like Teen Crisis TextLine, a free hotline primarily designed to reach young people age 13–22 in Louisiana at a critical stage in life (vialink.org/teen-text-program/). Both 988 Lifeline and Teen Crisis TextLine provide free, 24/7 crisis counseling via call, chat, or text, pairing clients with trained crisis counselors.

Crisis text line users are a high-risk, self-selected subgroup of Louisiana youth. Observed topic prevalence reflects help-seeking behavior, and some research suggests that crisis hotline help-seeking parallels trends in hospital administrative datasets during hurricane events [6]. For this analysis, the authors obtained anonymized crisis transcript data from VL for the Teen Crisis TextLine (covering approximately 457,128 Louisiana residents since 2018). Data included timestamps, crisis categorizations, self-reported demographics, geographic location data, and the anonymized text content of crisis communications (i.e., chat/text transcripts). Transcript data were preprocessed for topic modelling using standard text-analysis procedures, including lowercasing words, removing punctuation and stop words, and lemmatization [34]. Each transcript was tokenized and treated as a single “document” for analysis [35].

### Tropical cyclone data

Tropical cyclones included any storm making Louisiana landfall between 2018–2023: Hurricane Barry (July 2019; Category 1), Tropical Storm Cristobal (June 2020), Hurricane Laura (August 2020; Category 4), Hurricane Delta (October 2020; Category 2), Hurricane Zeta (October 2020; Category 3), and Hurricane Ida (August 2021; Category 4). Each crisis conversation was assigned to the closest hurricane based on the fewest days between the landfall date and the crisis conversation date. Recovery windows were informed by disaster mental health frameworks documenting multi-phase psychological responses persisting well beyond the acute phase, including elevated symptoms among youth up to two years following hurricane exposure [19,20]. Eight mutually exclusive recovery periods were defined: During Hurricane (days −1 to +2 from landfall), 3–30 Days Post, 31–60 Days Post, 61–90 Days Post, 3–6 Months Post (days 91–180), 6–9 Months Post (days 181–270), 9–12 Months Post (days 271–365), and Beyond 1 Year (>365 days). For conversations after 1-year the attribution of observed patterns to hurricane exposure becomes increasingly uncertain at these intervals, and findings from this period should be interpreted with appropriate caution.

## Methods

### Structural topic modelling

We employed Structural Topic Modelling (STM), an unsupervised machine learning approach that identifies latent thematic patterns in large text corpora and does not require pre-labeled topics [35,36]. This method builds upon Latent Dirichlet Allocation (LDA) [30,37], but unlike standard LDA, STM allows for inclusion of document-level covariates, such as the date of conversation, making it ideal for understanding how help-seeking behaviors and crisis concerns vary across temporal differences post-tropical cyclone. Other studies have used STM to examine temporal changes, such as mental health for pre/post celebrity suicides [38] and dimensions of religion [39]

### Corpus construction and text preprocessing

Text preprocessing followed established best practices for computational text analysis [40]. Preprocessing included: (1) tokenization and initial filtering, converting text to lowercase and removing punctuation, numbers, and special characters; (2) stopword removal using standard English stopwords combined with high-frequency terms lacking substantive meaning (e.g., counselor names, system artifacts, platform-specific terminology); (3) lemmatization for morphological normalization, ensuring semantically equivalent terms were consolidated; and (4) n-gram detection to preserve multi-word expressions that are especially useful for describing complex crisis-related concepts (e.g., “self-harm,” “panic attack,” “suicidal thoughts”). To refine vocabulary and reduce dimensionality, we implemented term filtering based on Term Frequency-Inverse Document Frequency (TF-IDF), retaining terms that are relatively distinctive within specific documents and informative across the entire corpus. Each transcript was tokenized and treated as a single “document” for analysis [35].

### Structural topic model specification

The number of topics (*K*) was selected by evaluating candidate values (*K* = 5, 7, 10, 12, 15, 18, 20, 25) against established diagnostics: held-out likelihood, residual dispersion, semantic coherence, and exclusivity [36,41]. We selected K = 12 based on diagnostic metrics and close reading of documents representative of each topic. The final 12-topic model demonstrated adequate fit, with a mean semantic coherence of −51.27 and a mean exclusivity of 9.28. Individual topic diagnostics ranged from −32.86 to −84.71 for semantic coherence and 8.68 to 9.95 for exclusivity (S1 Table). The STM prevalence model estimates conditional associations between document metadata and expected topic proportions [35,36]. The model specification was:


prevalence~hurricane_period+concurrent_crisis

Concurrent crisis context was operationalized as a binary indicator (0/1) distinguishing conversations embedded with tropical cyclones that occurred during the COVID-19 pandemic period from those that occurred before, capturing the co-occurrence of hurricane exposure with pandemic-related stressors.

### Topic interpretation

Topic labels were assigned using multiple word-ranking outputs combined with qualitative examination of representative high-proportion transcripts [36]. Two complementary word-ranking methods were used: probability, which ranks words by their likelihood of occurrence within a topic (identifying the most common words that define the topic’s core vocabulary); and FREX (Frequency-Exclusivity), which ranks words using a weighted harmonic mean of word frequency within a topic and exclusivity to that topic, identifying terms that are both common in the focal topic and rare in other topics [35,42]. This dual approach surfaces high-frequency terms that anchor the topic alongside distinctive terms that differentiate it from other topics. Topics were grouped into broader thematic domains for interpretation and analysis.

### Additional statistical analysis

Covariate effects on topic prevalence were estimated using the estimateEffect() function in the STM package [36], which implements a regression-based approach where document-level topic proportions serve as the outcome variable. This method of composition accounts for measurement uncertainty in topic assignments and the compositional nature of topic proportions. The hurricane recovery period was modeled as a categorical variable, with the 3–30 days post-hurricane period as the reference category. Confidence intervals were calculated using the default simulation-based approach with 500 draws. Effects were considered statistically significant at p < .05 (i.e., 95% confidence interval excluding zero)

## Results

### Descriptive statistics

A total of 2,149 crisis text conversations among Louisiana youth were recorded on the Teen Crisis TextLine from 2018 to 2023 (Table 1). The analytic sample comprised 1,335 unique individuals, of whom 67.7% (n = 904) contacted the line only once and 7.0% (n = 93) contacted five or more times. Among those with hurricane exposure (n = 1,039), only 45 individuals (4.3%) appeared across multiple distinct storm events. Hurricane Ida (Category 4) accounted for nearly 60% of the conversations (n = 1288), followed by Zeta (18.5%, n = 398), and Barry (17.5%, n = 377). The remaining hurricanes (Cristobal, Laura, and Delta) accounted for a smaller proportion (Table 1). Sample sizes ranged from landfall (n = 43) to beyond 12 months post-hurricane (n = 714). Approximately three-quarters of records (n = 1,591; 74%) occurred during compound disaster conditions (hurricane + COVID-19), while the remaining 26% (n = 558) occurred during single hurricane events (Table 1). The crisis text data were de-identified by VIA LINK prior to transfer, with all direct identifiers (including phone numbers) removed and replaced with a unique hashed person identifier.

**Table 1:** Sample Characteristics.

Characteristic	N	%
**Total Crisis Conversations**	**2,149**	**100.0**
***Hurricane Recovery Period***		
During Hurricane (landfall ± 3 days)	43	2.0
3–30 Days Post-Hurricane	112	5.2
31–60 Days Post-Hurricane	134	6.2
61–90 Days Post-Hurricane	142	6.6
3–6 Months Post-Hurricane	401	18.7
6–9 Months Post-Hurricane	387	18.0
9–12 Months Post-Hurricane	216	10.1
Beyond 12 Months Post-Hurricane^*b*^	714	33.2
***Disaster Context***		
Compound Disaster (Hurricane + COVID-19)	1,591	74.0
Single Disaster (Hurricane Only, Pre-COVID)	558	26.0
***Closest Hurricane at Time of Contact***		
Hurricane Ida (August 29, 2021; Category 4)	1,288	59.9
Hurricane Zeta (October 28, 2020; Category 3)	398	18.5
Hurricane Barry (July 13, 2019; Category 1)	377	17.5
Tropical Storm Cristobal (June 7, 2020)	48	2.2
Hurricane Laura (August 27, 2020; Category 4)ᵃ	25	1.2
Hurricane Delta (October 9, 2020; Category 2)ᵃ	13	0.6

*Note: Data represent crisis text conversations from the VIA LINK Teen Crisis TextLine serving Louisiana youth (2018–2023). Hurricane recovery periods are mutually exclusive and calculated from each conversation’s timestamp relative to the closest hurricane landfall date. Compound disaster exposure reflects conversations occurring during the COVID-19 pandemic period (March 2020 onward). Hurricane assignment follows a “closest hurricane” algorithm based on temporal proximity at time of contact.* ᵃ*Insufficient sample size for individual hurricane analysis; these storms occurred during a compressed 2020 hurricane season with four named storms within five months.*

*b Bounded by study end date (September 30, 2023). Observed range: over 366 days (median = ~19 months post-hurricane).*

### Topic model results

A 12-topic STM was selected based on model diagnostics (mean semantic coherence = −51.3; mean exclusivity = 9.3) and qualitative review of representative documents (S1 Table). Topics were organized into four substantive domains based on content analysis of high-probability words and associated texts (Table 2, S1 Fig). Topic prevalences ranged from approximately 4% (Louisiana Services) to 13.4% (Crisis Hotline Protocol). Topic co-occurrence analysis indicated that crisis text conversations typically focus on a single primary concern rather than addressing multiple issues simultaneously (S1 Fig). Notably, Suicide Ideation & Self-Harm (Topic 4) shows negative correlations with most other topics, suggesting that when youth discuss suicidal ideation, conversations remain focused on crisis content rather than diffusing across other stressors (S1 Fig, S2 Fig).

**Table 2:** Structural Topic Model Results: Topic Characteristics, Temporal Classification, and Representative Quotes.

#	Topic Label	Top FREX Terms		%	Peak Period	Wave	Example Quote
***Crisis Topics***							
4	Suicide Ideation & Self-Harm	cut, suicide, thought, promise, kill, act, part	fake, blade, razor, overdose, pill, useless, promise	8.3	61–90 Days	Wave 2	*“I’m suicidal... I’ve tried pills and other stuff... Everyday”*
9	Grief & Panic	baby, grandma, breathing, calm, doctor, panic attack, sense	patience	6.5	3–6 Months	Wave 2	*“I feel like I have a panic attack... my anxiety is making my thoughts think about something I don’t want to think about”*
12	Abuse & Safety Concerns	report, abuse, mother, identify, relationship, threaten, situation	identify, mandated, report, verbal	4.8	9–12 Months	Wave 3	*“She yelled and cursed at me when she found out that I told my teacher I wanted to cut myself.”*
***Coping Topics***							
2	Anxiety & Coping Skills	cope, journal, skill, anxiety, write, quit, music	quit, strategy, technique, journal, therapeutic, spiral	10.3	>12 Months	Wave 3	*“None of my tactics are helping me combat the feeling I have... I have a phobia of dentists and doctors.”*
6	Sleep & Self-Care	watch, sleep, eat, recent, night, gotcha, rest	Saturday, eaten	10.6	61–90 Days	Wave 1–2	*“I just feel alone all the time... I would kind of like to just talk about my feelings tonight.”*
***Stressor Topics***							
3	Academic Stress	class, test, grade, college, study, imagine, motivation	test, oof, study, senior, school_stress, class, college	7.3	31–60 Days	Wave 2	*“Midterms are coming up, and stressed. I just haven’t been motivated to do much.”*
7	Family Conflict	brother, mad, dad, fight, frustrated, sister	rant, piss, attitude, divorce	8.7	>12 Months	Wave 3	*“I recently moved out of a toxic home with my mom... nothing but fights and Chaos”*
10	Relationships & Dating	relationship, boyfriend, guy, friend, trust, date, hurt	rumor, flirt, confront, closure, breakup, genuine	12.6	>12 Months	Wave 3	*“I think I ruined a relationship... I keep choosing to do things I don’t even know if I actually want.”*
***Resource & Process Topics***							
8	Crisis Hotline Protocol	number, line, confidential, question, welcome, provide, send	homicide, demographic, age, dial	13.4	During	Wave 1	*“We provide confidential short-term crisis [support] and referrals for youth ages 12–22 throughout Louisiana.”*
11	Louisiana Crisis Services	Orleans, offer, money, suit, insurance, health, Friday	hammond, boulevard, outpatient, saint, shelter, charity	3.9	During	Wave 1	*“We lost all our food in the hurricane... Just one moment while I look for food assistance.”*
1	Follow-up & Reconnection	reach, text, apologize, delay, heard, wait, anytime	cancel, reach, delay, volume, apologize	4.5	>12 Months	Wave 3	*“Thanks for reaching out... I apologize for the delay... Please feel free to contact us back later.”*
5	Session Logistics	end, free, week, day, name, hour	actually, free, end, online, hour, binary	9.1	>12 Months	Wave 3	*“[Session ending]... You can contact us 24 hours a day, 7 days a week.”*

*Note: Topics were identified using a 12-topic Structural Topic Model (STM) with spectral initialization. FREX = Frequency-Exclusivity weighted terms. LIFT = words with the highest lift values, indicating terms most distinctive to each topic compared to the corpus overall; these reveal specific language patterns unique to each topic domain. Terms are translated from the stemmed to a readable form. Prevalence (%) = mean topic proportion across all N = 2,149 documents. Peak Period = recovery period with the highest mean topic prevalence. Wave classification: Wave 1 (Immediate) = During Hurricane through 30 days; Wave 2 (Delayed) = 31–90 days; Wave 3 (Long-term) = 3 + months post-hurricane. Example quotes are representative high-loading texts from each topic, with counselor names removed and minor edits for clarity [indicated in brackets].*

### Three waves of post-hurricane response

**Fig 1:**
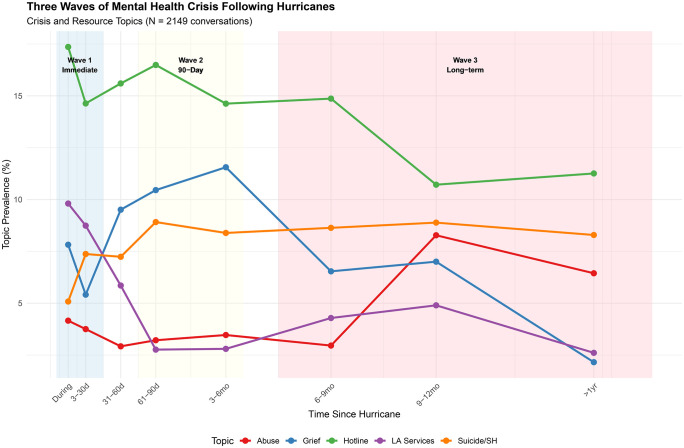
Topic prevalence trajectories illustrating the temporal dynamics of crisis and resource-seeking behaviors following hurricane exposure (N = 2,149 crisis text conversations).

Topics exhibited distinct temporal trajectories consistent with a three-wave pattern that was derived post hoc from observed peak prevalence patterns across eight recovery periods and as a descriptive organizing framework (Fig 1, S2 Table, S3 Fig). Classification of topics by peak wave revealed: three topics peaked during Wave 1/Immediate (Crisis Hotline Protocol, Louisiana Services, Sleep & Self-Care), three topics peaked during Wave 2/Delayed (Academic Stress, Suicide Ideation & Self-Harm, Grief & Panic), and six topics peaked during Wave 3/Long-term (Follow-up & Reconnection, Anxiety & Coping Skills, Session Logistics, Family Conflict, Relationships & Dating, Abuse & Safety Concerns).

**Wave 1 (Immediate: During Hurricane–30 Days).** Resource-seeking dominated the immediate response. Crisis Hotline Protocol (17.4%) and Louisiana Services (9.8%) peaked during active storms. Sleep & Self-Care showed a sustained plateau across the early recovery window, with nearly identical prevalence at 3–30 days (14.0%) and 61–90 days (14.2%), followed by a significant decline in long-term recovery (6.9% at >12 months; β = −0.063, 95% CI [−0.095, −0.031]). Relative to the immediate post-hurricane reference period (3–30 days post-hurricane), Louisiana Services showed a significant decline across all subsequent periods (β range: −0.041 to −0.060, all p < .05) (S3 Table).

**Fig 2:**
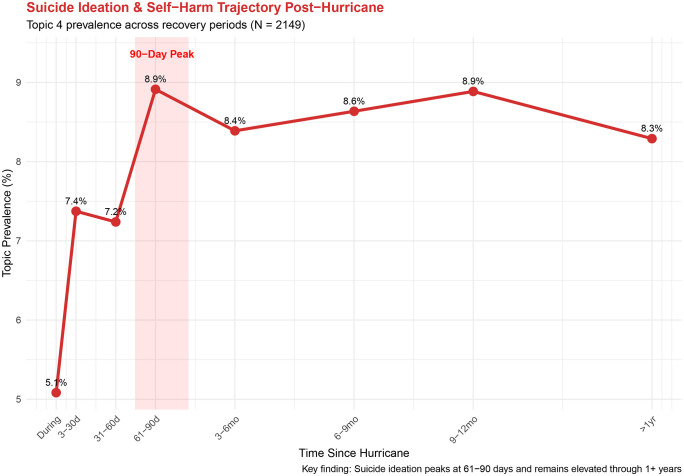
Exposure Temporal trajectory of Topic 4 (Suicide Ideation & Self-Harm) prevalence across eight mutually exclusive hurricane recovery periods (N= 2,149 crisis text conversations).

**Wave 2 (Delayed: 31–90 Days).** Relative to the immediate post-hurricane reference period (3–30 days post-hurricane), Grief & Panic showed significant elevation at 61–90 days (β = 0.048, 95% CI [0.004, 0.092]) and 3–6 months (β = 0.058, 95% CI [0.021, 0.094]). Academic Stress peaked at 31–60 days (10.4%), and Suicide Ideation & Self-Harm reached its highest prevalence at 61–90 days (8.9%) (Fig 2). While the Suicide Ideation & Self-Harm did not reach significance in the STM covariate model relative to the 3–30 day reference period, trajectory analysis comparing the during-hurricane baseline to the 61–90 day peak was statistically significant (t = 2.78, p = 0.006; Table 3). Suicide-related topic prevalence remained elevated throughout the recovery period (range 7.2-8.9% from 31 days through 12 months post-landfall), suggesting a sustained post-disaster crisis burden rather than a single discrete peak.

**Table 3:** Suicide Ideation Topic Trajectory with Hurricane Ida Sensitivity Analysis.

	Full Sample (N = 2,149)			Hurricane Ida Only (n = 1,288)		
**Recovery Period**	**n**	**Prev.**	**95% CI**	**n**	**Prev.**	**95% CI**
During Hurricane	43	5.1%	[3.3, 6.8]	12	5.5%	[1.8, 9.1]
3–30 Days Post	112	7.4%	[5.4, 9.4]	26	7.4%	[3.0, 11.8]
31–60 Days Post	134	7.2%	[5.6, 8.8]	39	9.5%	[6.0, 13.0]
61–90 Days Post	142	8.9%	[6.8, 11.0]	61	11.3%	[7.1, 15.5]
3–6 Months Post	401	8.4%	[7.3, 9.5]	130	8.9%	[6.8, 11.1]
6–9 Months Post	387	8.6%	[7.5, 9.8]	163	9.6%	[7.6, 11.6]
9–12 Months Post	216	8.9%	[7.5, 10.2]	143	9.5%	[7.7, 11.3]
>12 Months Post	714	8.3%	[7.5, 9.1]	714	8.3%	[7.5, 9.1]
***Statistical Comparison: Peak (61–90 Days) vs. Baseline (During Hurricane)***						
**Absolute change**	**+3.8 percentage points**			**+5.8 percentage points**		
Relative change	+74.5%			+105.5%		
Welch’s t-test	**t(153.7) = 2.78, p = 0.006**			**t(44.9) = 2.06, p = 0.046**		

*Note: Table presents Topic 4 (Suicide Ideation & Self-Harm) prevalence across hurricane recovery periods for the full sample and Hurricane Ida subsample. Prevalence represents the mean document-level topic proportion. 95% confidence intervals calculated using period-specific standard errors. The shaded row indicates peak prevalence at 61–90 days post-hurricane. Sensitivity analysis is restricted to Hurricane Ida (n = 1,288; 59.9% of the full sample), the most severe storm during the study period (Category 4 at landfall in Louisiana). The larger effect size in the Ida-only analysis (+5.8 percentage points (pp) vs. + 3.8 pp) demonstrates that Ida’s sample dominance provides statistical power rather than introducing bias. Welch’s t-test is used to account for unequal variances between recovery periods.*

Because we observed the largest text/call volume after Hurricane Ida, a supplementary analysis of records (n = 1,288) was performed, revealing a more pronounced suicide ideation trajectory (Table 3). Among Ida-exposed youth, Suicide Ideation & Self-Harm increased from 5.5% during the hurricane to 11.3% at 61–90 days post-landfall, representing a 5.8 percentage point increase that was statistically significant (t = 2.06, p = 0.046).

**Wave 3 (Long-Term: 3 + Months).** Relative to the reference period, Family Conflict increased significantly at 9–12 months (β = 0.038, 95% CI [0.001, 0.075]) and beyond 12 months (β = 0.034, 95% CI [0.002, 0.066]). Relationships & Dating peaked at more than 12 months (17.8%; β = 0.059, 95% CI [0.021, 0.097]). Abuse & Safety Concerns showed delayed emergence, with significant increases at 9–12 months (β = 0.039, 95% CI [0.008, 0.071]) and beyond 12 months (β = 0.030, 95% CI [0.005, 0.055]). Sleep & Self-Care showed a significant decline beyond 12 months (β = −0.063, 95% CI [−0.095, −0.031]).

### Concurrent crisis effects

**Fig 3:**
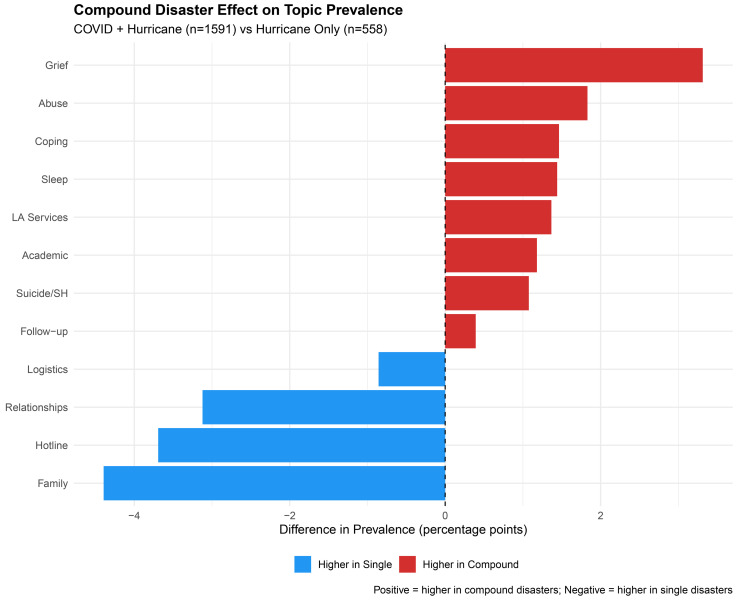
Comparison of topic prevalence between concurrent disasters (hurricane occurring during the COVID-19 pandemic, n = 1,591) versus single disasters (hurricane only, pre-COVID, n = 558). Bar length represents the difference in topic prevalence (percentage points), with positive values indicating higher prevalence in compound disaster conditions. Single disaster reference group = Hurricane Barry (July 2019).

Concurrent disasters demonstrated differential topic prevalence across all four domains compared to single-hurricane events (Fig 3, S4 Table). Within Crisis topics, Grief & Panic showed the largest compound amplification (+3.3 pp), followed by Abuse & Safety Concerns (+1.8 pp) and Suicide Ideation (+1.1 pp). Within Stressor topics, Family Conflict (−4.4 pp) and Relationships (−3.1 pp) were more prevalent in single-disaster contexts, potentially reflecting reduced interpersonal contact during pandemic conditions.

## Discussion

This study provides novel evidence that youth crisis help-seeking following hurricanes follows a dynamic, multi-phase recovery trajectory rather than a single, short-term surge in mental health needs. Leveraging anonymized crisis text conversations from Louisiana youth, a novel real-time data source, and structural topic modeling (STM), which uniquely enables assessment of covariate effects on topic prevalence [39], we identified 12 distinct crisis topics and documented a systematic “three waves” pattern of post-disaster psychological response: immediate resource-seeking and safety stabilization (~30 days), delayed emergence of psychological distress including grief, academic stress, and suicidal ideation (~3 months), and a later shift toward interpersonal conflict, relationship stress, and abuse-related concerns during prolonged recovery (6–12 months). Additionally, concurrent disaster exposure during the COVID-19 pandemic amplified several distress-related crisis concerns, suggesting that co-occurring crises may intensify youth mental health vulnerability. Together, these findings advance current understanding of how youth mental health needs evolve across the disaster recovery continuum and highlight critical windows for the timing and targeting of post-disaster crisis interventions.

In general, the three-wave pattern identified in this study contextualizes existing theoretical frameworks of post-disaster psychological response. Classic models of disaster mental health have emphasized an initial “heroic” phase followed by a “honeymoon” period and subsequent “disillusionment” [43,44]. Our findings provide empirical support for these phases and offer greater temporal granularity for youth populations. Specifically, our results illustrate that help-seeking behaviors do not necessarily decline in the honeymoon/heroic phase; rather, young people engage in immediate help-seeking for information, referrals, and resources. Indeed, the predominance of resource-seeking topics (e.g., Crisis Hotline Protocol, Louisiana Services) during active hurricanes and the immediate aftermath aligns with the acute survival orientation described in disaster response literature [45–47]. Our findings suggest that youth seek a social safety net during the immediate recovery period, seeking information, resources, and referral services to address their needs.

The second wave of help-seeking was characterized by the delayed emergence of acute crisis topics, such as suicide ideation, self-harm, grief, and panic. This delayed peak is consistent with prior longitudinal research on Hurricane Katrina, showing that PTSD symptoms may emerge or persist in populations months after exposure, partially explained by ongoing stressors, which can erode resources and resilience [48]. Among youth, grief can manifest and contribute to a host of mental health concerns, including post-traumatic stress [49], and prior work suggests disaster grief among youth emerges from a variety of intersecting stressors, including loss of a loved one, family disruption, and interpersonal strain [49–51]. Emerging research from COVID-19 further shows that pandemic-related bereavement was associated with elevated risks of prolonged grief, depression, and suicidal ideation, particularly around the loss of loved ones and the absence of mourning rituals [52]. COVID-related loss has also been associated with increased vulnerability to mental health issues in children and adolescents [53,54]. In parallel, disaster and pandemic-driven interpersonal strain and poor social support increase the risk of chronic post-traumatic stress among youth and young people [20], requiring targeted, stepped-up care interventions that extend into prolonged recovery periods [51].

Concerningly, conversations that mentioned suicide and self-harm did not decrease following the delayed peak, but rather remained elevated for over a year after exposure, suggesting delayed emergence of prolonged crises among young people. Prior work has highlighted similar trends, in which exposed populations exhibit an initial decline in suicidality, followed by extended increases attributed to ongoing stressors, such as slow disaster recovery and interpersonal concerns [55,56]. These results likely highlight the dynamic and sustained psychological health impacts associated with tropical cyclone exposure in combination with the COVID-19 pandemic among young people, contributing to increased suicide-related conversations over a year after exposure. Integration of suicide prevention into disaster recovery efforts is essential, and additional research is needed to identify effective, evidence-based, and culturally tailored interventions to reduce disaster-related suicidality among youth [51,57]. Crisis lines serve as a safety-net for youth with unaddressed mental health needs, and more research is needed into how these lines can be intake and referral points for local support.

The observed third wave, characterized by interpersonal stressors that emerge more than 3 months after a hurricane, suggests that disasters have cascading effects on family systems and social relationships. This finding aligns with ecological models that emphasize that youth mental health is embedded in family and community systems [58,59] and that stress within or disruptions to these systems can adversely influence youth [60]. The delayed emergence of Abuse & Safety Concerns may also reflect increased household stress, economic strain, or disrupted protective factors of family and community systems during prolonged recovery periods [60–62]. Prior work has emphasized that colliding economic and interpersonal stressors may explain and contribute to increased reports of child abuse during long-term disaster recovery [61–63]. As such, slow disaster response (e.g., delayed funding) and limited resource availability (e.g., cramped housing, limited transportation) following disaster exposure may contribute to interpersonal strain, abuse, and the emergence of delayed, high-severity crises among youth [61–63]. The persistence of crises related to family and interpersonal stressors over a year after exposure highlights critical long-term implications for youth health and well-being. Young people have limited developmental capacity to process and cope with traumatic life events, such as a tropical cyclone [64]. Social support emerges consistently as a protective factor for youth, buffering against the long-term negative psychological health impacts associated with disaster exposure [20,64,65]. The high prevalence of crisis conversations about interpersonal stressors and family conflicts a year after exposure points to the ongoing need for psychosocial support resources, specifically for young people and families with young children following large-scale disasters [51,66]. Future work should investigate the co-evolution of economic and interpersonal stressors following disaster exposure to inform effective upstream interventions that address resource strain and prioritize interpersonal safety in the immediate response phase and throughout the stress of long-term recovery.

The differential topic patterns observed between compound and single disaster contexts suggest that COVID-19 pandemic conditions may have amplified certain crisis pathways [67]. The higher prevalence of grief and panic during co-occurring disasters may reflect the cumulative psychological toll of simultaneous threats, including pandemic-related losses and heightened health anxiety [68]. Conversely, the lower prevalence of family conflict and relationship issues in concurrent disasters could reflect reduced interpersonal contact during pandemic restrictions or alternatively, a prioritization of acute crisis concerns over interpersonal stressors. Notably, compound disaster exposure was associated with higher suicide ideation prevalence, consistent with evidence that multiple disaster exposures compound mental health risk [5]. Hurricane Ida heavily influenced this dataset, accounting for nearly 60% of conversations. While the suicide ideation trajectory finding remains robust across sensitivity analyses, other topic-level patterns may uniquely reflect Ida’s specific context (severity, concurrent COVID-19, and south Louisiana’s geographic damage). Therefore, replication across varied hurricane events and regions is necessary before generalizing these patterns to broader multi-hurricane dynamics.

### Implications

While youth may show signs of initial distress in the aftermath of a disaster, most children are expected to recover with support. Yet a significant minority have lingering mental health problems and are in need of additional support to recover and function normally [69,70]. To date, the stepped-care approach to post-disaster recovery is recommended for both children and adults [51], with community-level interventions in the first days and weeks after the disaster, followed by increasingly intensive, targeted interventions for ongoing distress. This approach requires effective screening and triage practices to provide an appropriate level of care [71]. Crisis hotlines, such as Teen Crisis TextLine and 988, can provide initial triage, screening, and universal care, and facilitate stepped-up care through referrals to individual therapy or high-intensity specialty services. Our findings inform this stepped care framework by identifying temporal windows during which triage should be intensified (e.g., 61–90 days and after 9 months). In addition, data from these hotlines can serve as a real-time surveillance tool to monitor evolving mental health needs across the recovery continuum, informing the timing and targeting of stepped care interventions [6,7].

### Limitations

Several limitations should be considered when interpreting these findings. First, crisis text line users represent a self-selected population of help-seekers and may not be representative of all hurricane-affected youth. Those experiencing the most severe crises may not reach out to text-based services, while those with milder distress may not meet the threshold for crisis contact. Second, the geographic scope was limited to Louisiana, and patterns may differ in other Gulf Coast states or regions with different demographic compositions, hurricane histories, or crisis service infrastructures. Third, the uneven distribution of conversations across recovery periods, with only 2% occurring during active hurricanes and 33% occurring more than 12 months after the hurricane, limits the statistical power of some comparisons. The small sample size during hurricanes may reflect reduced cell service, evacuation, or competing survival priorities rather than lower crisis need. Fourth, topic modeling involves interpretive choices in labeling and categorization. While we employed multiple word metrics (e.g., FREX) and examined representative documents to validate labels, topic boundaries may span multiple themes. In addition, effects were estimated without correction for multiple testing, and therefore some findings may represent chance associations. However, this analysis does not claim that observed patterns are causally attributable to hurricane exposure; rather, our analysis identified temporal trends in crisis help-seeking behavior that co-occur with hurricane events. Although some individuals contacted the line on multiple occasions, the majority of the sample (~68%) had only a single conversation, and the 61–90 day recovery window had the lowest repeat-contact rate of any post-hurricane period (~1.5 conversations per person). High-frequency users were not disproportionately concentrated in this window (34.9% vs. a sample-wide average of 42.7%), and the delayed suicide ideation peak persisted when analyses were restricted to first contacts only, suggesting key findings are not driven by repeat-contact bias. Another key limitation is that the beyond-one-year group spans up to ~2 years post-landfall, and attributing observed patterns to hurricane exposure becomes increasingly uncertain at these intervals, given the potential influence of unrelated life events and broader societal factors. Sensitivity analyses treating beyond-one-year records as a background reference period found that suicide-related topic prevalence remained consistently elevated throughout recovery relative to this baseline, suggesting that crisis burden persisted well into the long-term recovery phase rather than resolving, though causal attribution at these time intervals cannot be established. Finally, we were unable to examine demographic moderators (e.g., age, race/ethnicity, gender identity, sexual orientation) due to inconsistent availability of these variables, limiting our ability to identify differential vulnerability within the youth population. For instance, age was available for approximately 66% of records; among those, 79% were under 18 and 21% were 18 or older, and both subgroups showed elevated suicide-related conversation rates at 61–90 days post-landfall (18.2% and 21.1%, respectively), though the substantial proportion of missing age data (~34%) precluded its inclusion as a covariate in the primary analysis.

## Conclusions

This study aimed to characterize the temporal dynamics of youth mental health help-seeking following hurricane exposure using anonymized crisis text conversations. Our analysis identified a three-wave pattern of post-disaster response: immediate resource-seeking, delayed crisis emergence, and long-term interpersonal stressors. Grief & Panic showed significant elevation at 61–90 days relative to the immediate post-hurricane period, while Suicide Ideation & Self-Harm exhibited a notable trajectory from 5.1% during hurricanes to 8.9% at 61–90 days (significant when compared to baseline), remaining elevated through 12 + months. Family Conflict and Abuse & Safety Concerns showed significant delayed emergence at 9 + months, suggesting cascading effects on family systems during prolonged recovery. Importantly, the majority of crisis conversations occurred during concurrent hurricane and COVID-19 conditions, highlighting how co-occurring disasters may intensify and prolong youth mental vulnerability. Pandemic-related disruptions, including social isolation, caregiver loss, economic strain, and school and community-based interruptions, likely worsened grief, anxiety, and crisis severity while limiting access to traditional protective supports. These temporal patterns align with stepped-care frameworks that recommend intensified screening during specific post-disaster intervals. Crisis text data offer a complementary surveillance tool for monitoring evolving youth mental health needs across the disaster recovery continuum, with implications for resource allocation and intervention timing following climate-related disasters.

## Supporting information

S1 Code R code used in this analysis. (R)

S1 Fig Topic Co-Occurrence Network: Network visualization of topic correlations across N = 2,149 crisis text conversations. Nodes represent the 12 topics identified by structural topic modeling, with node size proportional to overall topic prevalence and color indicating domain classification (red = Crisis, green = Coping, blue = Stressor, purple = Resources, orange = Process). Edges connect topics with absolute correlation coefficients |r| > 0.10, calculated from document-level topic proportions. Blue edges indicate positive correlations (topics that tend to co-occur within conversations); red edges indicate negative correlations (topics that are mutually exclusive). The predominance of negative correlations demonstrates that crisis text conversations typically focus on a single primary concern rather than addressing multiple issues simultaneously. Notably, Suicide Ideation & Self-Harm (Topic 4) shows negative correlations with most other topics, suggesting that when youth discuss suicidal ideation, conversations remain focused on crisis content rather than diffusing across other stressors. The only clusters of positive co-occurrence involve process-related topics (Logistics, Follow-up, Hotline), reflecting the administrative components that accompany substantive crisis discussions.
(DOCX)

S2 Fig Heatmap visualization of topic prevalence (%) for all 12 topics (rows) across 8 hurricane recovery periods (columns). Color intensity represents the magnitude of prevalence: blue indicates lower prevalence, red indicates higher prevalence. Numeric cell values display exact prevalence percentages. Topics ordered vertically by overall mean prevalence (highest at top).
(DOCX)

S3 Fig Faceted display of prevalence trajectories for all 12 topics identified by the Structural Topic Model (N = 2,149 crisis text conversations). Each panel displays the prevalence of a single topic (y-axis, %) across simplified recovery time periods (x-axis). Topics are arranged in a 4 × 3 grid and color-coded by domain: Crisis (red), Coping (green), Stressor (blue), and Resources/Process (gray). Shaded bands represent 95% confidence intervals. This comprehensive view reveals heterogeneous temporal patterns across topic domains: resource-seeking topics (Hotline, LA Services) decline over time, crisis topics (Suicide/SH, Grief) show delayed peaks, and interpersonal stressor topics (Relationships, Family) increase in the long-term recovery phase.
(DOCX)

S1 Table Structural Topic Model Selection and Diagnostics. (DOCX)

S2 Table Complete Temporal Dynamics: All Topic Prevalences Across Hurricane Recovery Periods. (DOCX)

S3 Table Statistically Significant Temporal Effects on Topic Prevalence from the Structural Topic Model. (DOCX)

S4 Table Compound Disaster Effects: Topic Prevalence by Disaster Context. (DOCX)
