# Supplementary material for: Temporal dynamics of teen crisis help-seeking following hurricanes: A structural topic model analysis
Source: PLOS Digit Health. 2026 May 12;5(5):e0001393. doi: 10.1371/journal.pdig.0001393 (PMC13166961; doi:10.1371/journal.pdig.0001393)
Supplement: S1 Fig — Nodes represent the 12 topics identified by structural topic modeling, with node size proportional to overall topic prevalence and color indicating domain classification (red = Crisis, green = Coping, blue = Stressor, purple = Resources, orange = Process). Edges connect topics with absolute correlation coefficients |r| > 0.10, calculated from document-level topic proportions. Blue edges indicate positive correlations (topics that tend to co-occur within conversations); red edges indicate negative correlations (topics that are mutually exclusive). The predominance of negative correlations demonstrates that crisis text conversations typically focus on a single primary concern rather than addressing multiple issues simultaneously. Notably, Suicide Ideation & Self-Harm (Topic 4) shows negative correlations with most other topics, suggesting that when youth discuss suicidal ideation, conversations remain focused on crisis content rather than diffusing across other stressors. The only clusters of positive co-occurrence involve process-related topics (Logistics, Follow-up, Hotline), reflecting the administrative components that accompany substantive crisis discussions. (DOCX) [file pdig.0001393.s002.docx]

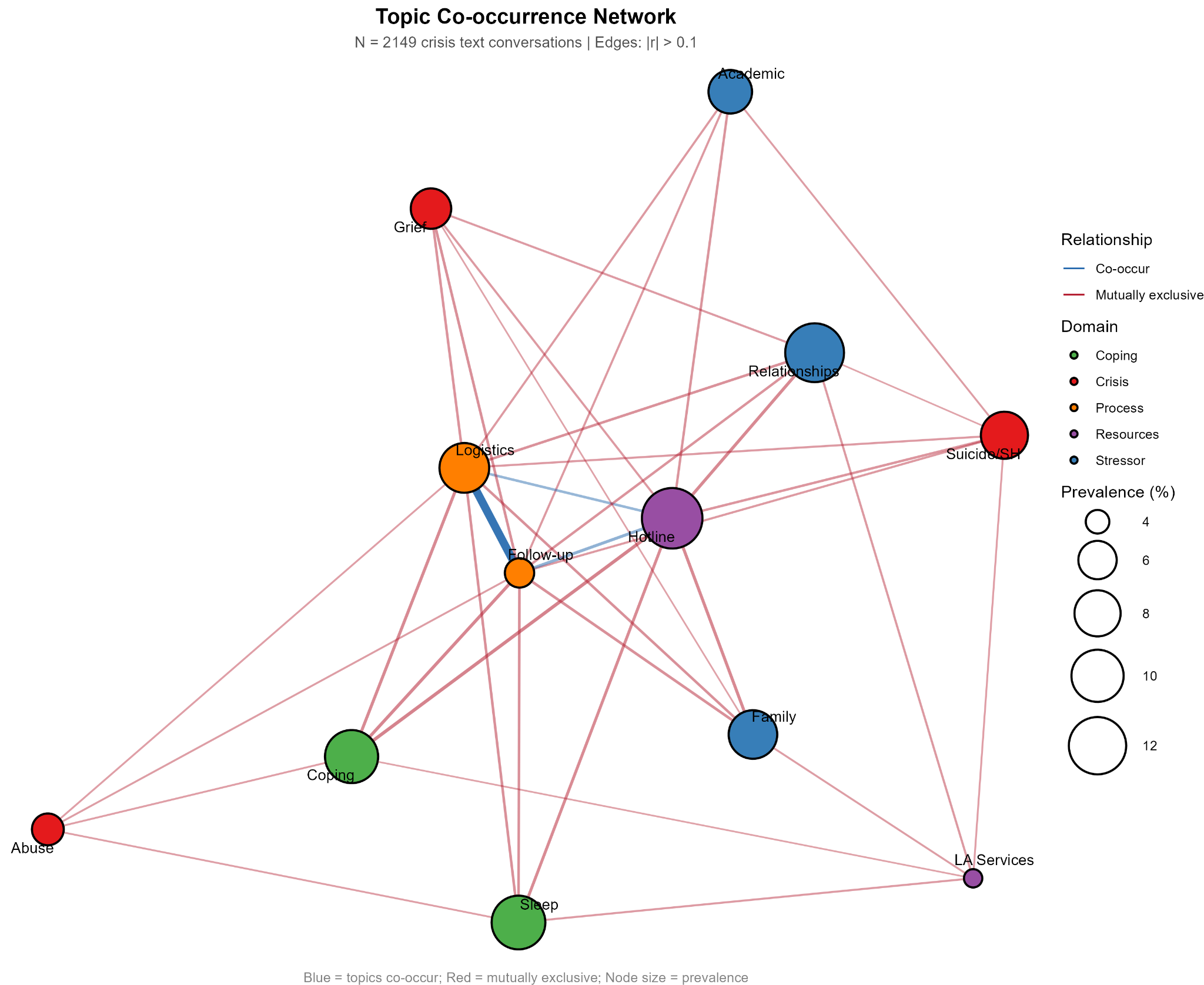


Figure S1. Topic Co-Occurrence Network: Network visualization of topic correlations across N = 2,149 crisis text conversations. Nodes represent the 12 topics identified by structural topic modeling, with node size proportional to overall topic prevalence and color indicating domain classification (red = Crisis, green = Coping, blue = Stressor, purple = Resources, orange = Process). Edges connect topics with absolute correlation coefficients |r| > 0.10, calculated from document-level topic proportions. Blue edges indicate positive correlations (topics that tend to co-occur within conversations); red edges indicate negative correlations (topics that are mutually exclusive). The predominance of negative correlations demonstrates that crisis text conversations typically focus on a single primary concern rather than addressing multiple issues simultaneously. Notably, Suicide Ideation & Self-Harm (Topic 4) shows negative correlations with most other topics, suggesting that when youth discuss suicidal ideation, conversations remain focused on crisis content rather than diffusing across other stressors. The only clusters of positive co-occurrence involve process-related topics (Logistics, Follow-up, Hotline), reflecting the administrative components that accompany substantive crisis discussions.
