# Supplementary material for: Temporal dynamics of teen crisis help-seeking following hurricanes: A structural topic model analysis
Source: PLOS Digit Health. 2026 May 12;5(5):e0001393. doi: 10.1371/journal.pdig.0001393 (PMC13166961; doi:10.1371/journal.pdig.0001393)
Supplement: S2 Fig — Color intensity represents the magnitude of prevalence: blue indicates lower prevalence, red indicates higher prevalence. Numeric cell values display exact prevalence percentages. Topics ordered vertically by overall mean prevalence (highest at top). (DOCX) [file pdig.0001393.s003.docx]

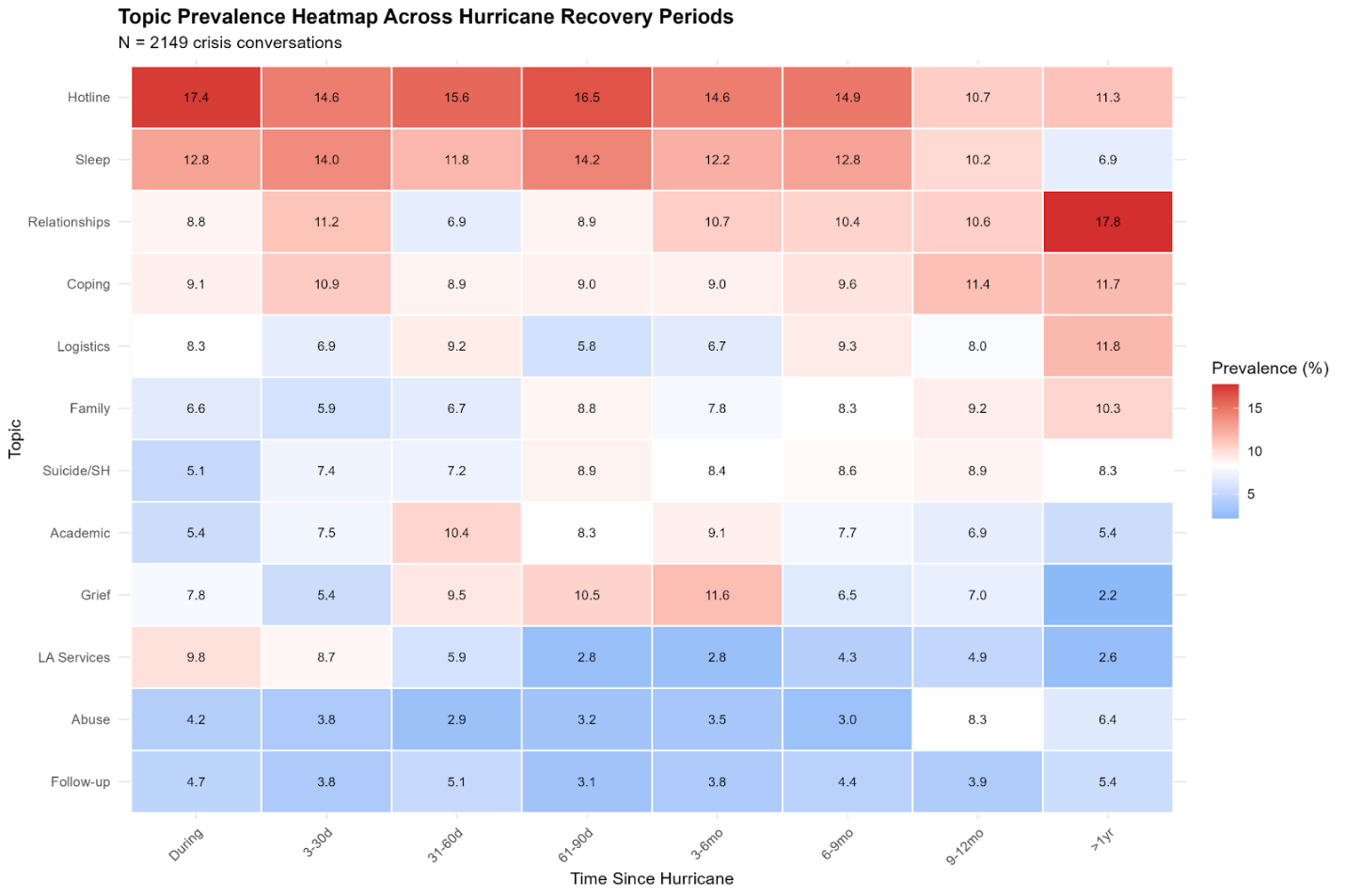


**Figure S2. Topic Prevalence Heatmap Across Hurricane Recovery Periods**

Heatmap visualization of topic prevalence (%) for all 12 topics (rows) across 8 hurricane recovery periods (columns). Color intensity represents the magnitude of prevalence: blue indicates lower prevalence, red indicates higher prevalence. Numeric cell values display exact prevalence percentages. Topics ordered vertically by overall mean prevalence (highest at top).
