# Supplementary material for: Temporal dynamics of teen crisis help-seeking following hurricanes: A structural topic model analysis
Source: PLOS Digit Health. 2026 May 12;5(5):e0001393. doi: 10.1371/journal.pdig.0001393 (PMC13166961; doi:10.1371/journal.pdig.0001393)
Supplement: S3 Fig — Each panel displays the prevalence of a single topic (y-axis, %) across simplified recovery time periods (x-axis). Topics are arranged in a 4 × 3 grid and color-coded by domain: Crisis (red), Coping (green), Stressor (blue), and Resources/Process (gray). Shaded bands represent 95% confidence intervals. This comprehensive view reveals heterogeneous temporal patterns across topic domains: resource-seeking topics (Hotline, LA Services) decline over time, crisis topics (Suicide/SH, Grief) show delayed peaks, and interpersonal stressor topics (Relationships, Family) increase in the long-term recovery phase. (DOCX) [file pdig.0001393.s004.docx]

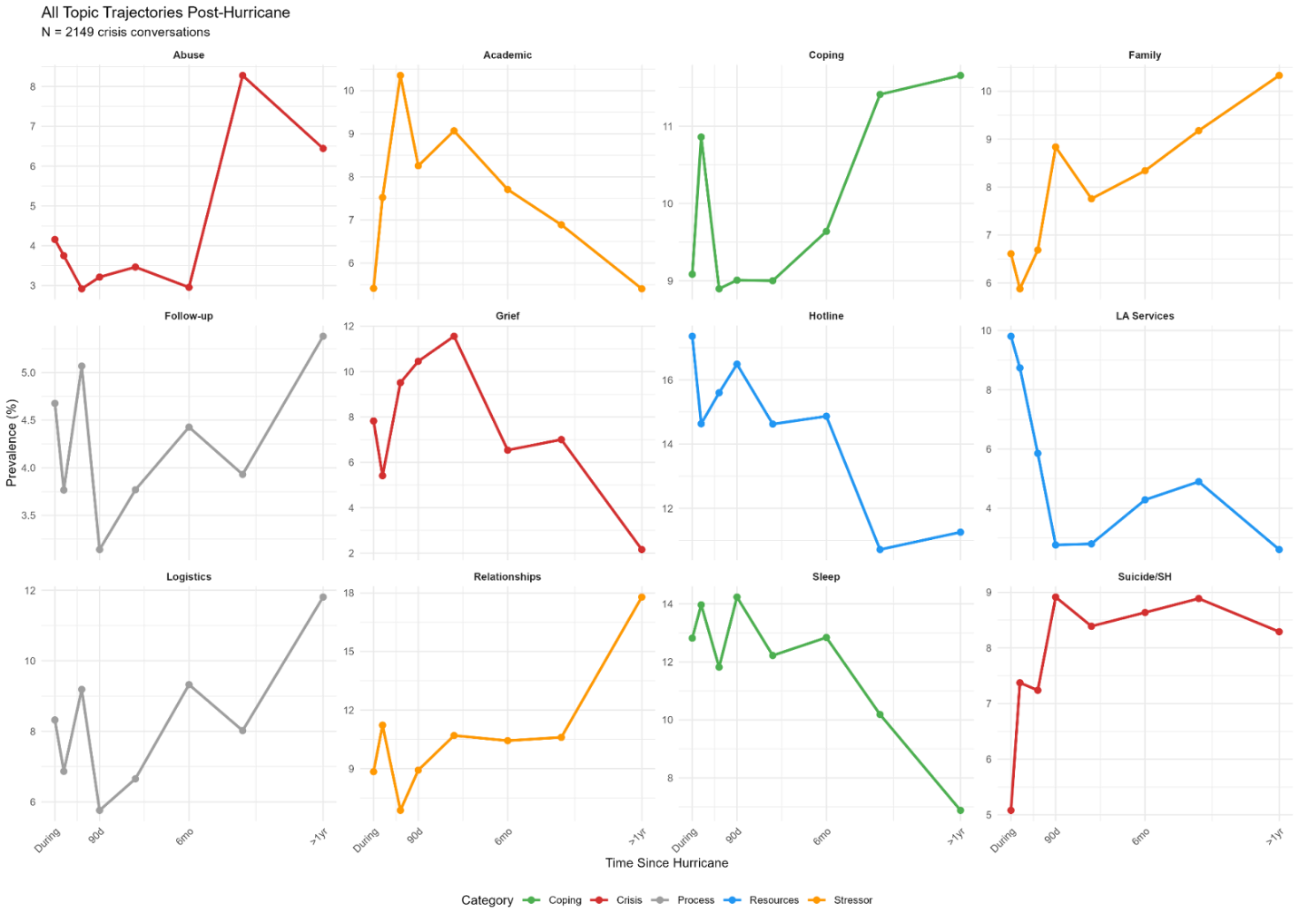


**Figure S3. Complete Topic Trajectory Panel: All 12 Topics Across Hurricane Recovery Periods**

Faceted display of prevalence trajectories for all 12 topics identified by the Structural Topic Model (N = 2,149 crisis text conversations). Each panel displays the prevalence of a single topic (y-axis, %) across simplified recovery time periods (x-axis). Topics are arranged in a 4×3 grid and color-coded by domain: Crisis (red), Coping (green), Stressor (blue), and Resources/Process (gray). Shaded bands represent 95% confidence intervals. This comprehensive view reveals heterogeneous temporal patterns across topic domains: resource-seeking topics (Hotline, LA Services) decline over time, crisis topics (Suicide/SH, Grief) show delayed peaks, and interpersonal stressor topics (Relationships, Family) increase in the long-term recovery phase.
