# Supplementary material for: Temporal dynamics of teen crisis help-seeking following hurricanes: A structural topic model analysis
Source: PLOS Digit Health. 2026 May 12;5(5):e0001393. doi: 10.1371/journal.pdig.0001393 (PMC13166961; doi:10.1371/journal.pdig.0001393)
Supplement: S1 Table — (DOCX) [file pdig.0001393.s005.docx]

**S1 Table.** Structural Topic Model Selection and Diagnostics

| **Model Selection: Candidate Topic Numbers (K = 5–25)** | | | | |
| --- | --- | --- | --- | --- |
| **K** | **Semantic Coherence** | **Exclusivity** | **Held-Out Likelihood** | **Combined Score** |
| 5 | -46.06 | 9.07 | -5.88 | 0.370 |
| 7 | -34.95 | 8.94 | -5.92 | 0.333 |
| 10 | -45.69 | 9.08 | -5.83 | 0.453 |
| **12*** | **-42.77** | **9.34** | **-5.79** | **0.695** |
| 15 | -48.49 | 9.39 | -5.80 | 0.644 |
| 18 | -57.98 | 9.48 | -5.74 | 0.693 |
| 20 | -56.75 | 9.48 | -5.73 | 0.721 |
| 25 | -66.68 | 9.58 | -5.74 | 0.646 |
| **Final Model: Topic-Level Diagnostics (K = 12)** | | | | |
| **Topic** | **Label** | **Semantic Coherence** | **Exclusivity** | |
| 1 | Follow-up/Check-in | -84.71 | 9.93 | |
| 2 | Coping Strategies | -39.05 | 9.12 | |
| 3 | Academic Stress | -32.86 | 8.68 | |
| 4 | Suicide/Self-Harm | -41.08 | 9.41 | |
| 5 | Conversation Logistics | -67.62 | 9.95 | |
| 6 | Sleep Disturbance | -40.94 | 9.50 | |
| 7 | Family Conflict | -52.09 | 8.75 | |
| 8 | Crisis Hotline Navigation | -44.35 | 9.53 | |
| 9 | Grief/Loss | -37.27 | 8.95 | |
| 10 | Relationship Issues | -34.75 | 8.97 | |
| 11 | Louisiana Services | -78.21 | 9.29 | |
| 12 | Abuse/Trauma | -62.26 | 9.30 | |

*Note.* N = 2,149 crisis text conversations. Model selection used searchK with spectral initialization (seed = 12345). Semantic coherence measures within-topic word co-occurrence (higher = more interpretable). Exclusivity measures between-topic distinctiveness (higher = more unique). Held-out likelihood assesses predictive validity. Combined score = normalized average of semantic coherence, exclusivity, and held-out likelihood (range 0–1). Final model: mean semantic coherence = -51.27; mean exclusivity = 9.28.

* Selected model. K = 12 chosen for high combined score (0.695) with superior semantic coherence versus higher-K models.
