# Supplementary material for: Temporal dynamics of teen crisis help-seeking following hurricanes: A structural topic model analysis
Source: PLOS Digit Health. 2026 May 12;5(5):e0001393. doi: 10.1371/journal.pdig.0001393 (PMC13166961; doi:10.1371/journal.pdig.0001393)
Supplement: S2 Table — (DOCX) [file pdig.0001393.s006.docx]

## **S2 Table**. Complete Temporal Dynamics: All Topic Prevalences Across Hurricane Recovery Periods

| **Topic** | **During** | **3–30d** | **31–60d** | **61–90d** | **3–6mo** | **6–9mo** | **9–12mo** | **>12mo** |
| --- | --- | --- | --- | --- | --- | --- | --- | --- |
| **Sample Size (n)** | **43** | **112** | **134** | **142** | **401** | **387** | **216** | **714** |
| ***Crisis Topics*** | | | | | | | | |
| **Suicide/Self-Harm** | 5.1 | 7.4 | 7.2 | **8.9** | 8.4 | 8.6 | 8.9 | 8.3 |
| Grief & Panic | 7.8 | 5.4 | 9.5 | 10.5 | **11.6** | 6.5 | 7.0 | 2.2 |
| Abuse & Safety | 4.2 | 3.8 | 2.9 | 3.2 | 3.5 | 3.0 | **8.3** | 6.4 |
| ***Coping Topics*** | | | | | | | | |
| Anxiety & Coping | 9.1 | 10.9 | 8.9 | 9.0 | 9.0 | 9.6 | 11.4 | **11.7** |
| Sleep & Self-Care | 12.8 | 14.0 | 11.8 | **14.2** | 12.2 | 12.8 | 10.2 | 6.9 |
| ***Stressor Topics*** | | | | | | | | |
| Academic Stress | 5.4 | 7.5 | **10.4** | 8.3 | 9.1 | 7.7 | 6.9 | 5.4 |
| Family Conflict | 6.6 | 5.9 | 6.7 | 8.8 | 7.8 | 8.3 | 9.2 | **10.3** |
| Relationships | 8.8 | 11.2 | 6.9 | 8.9 | 10.7 | 10.4 | 10.6 | **17.8** |
| ***Resource & Process Topics*** | | | | | | | | |
| Hotline Protocol | **17.4** | 14.6 | 15.6 | 16.5 | 14.6 | 14.9 | 10.7 | 11.3 |
| LA Crisis Services | **9.8** | 8.7 | 5.9 | 2.8 | 2.8 | 4.3 | 4.9 | 2.6 |
| Follow-up | 4.7 | 3.8 | 5.1 | 3.1 | 3.8 | 4.4 | 3.9 | **5.4** |
| Session Logistics | 8.3 | 6.9 | 9.2 | 5.8 | 6.7 | 9.3 | 8.0 | **11.8** |

*Note: Values represent topic prevalence (%) within each mutually exclusive recovery period (N = 2,149 total conversations). Blue shading indicates peak prevalence for each topic row. Orange shading highlights the Suicide Ideation & Self-Harm peak at 61–90 days post-hurricane, the primary outcome. Sample sizes per period are shown in the first data row. Topics organized by domain: Crisis (topics directly related to acute psychological distress), Coping (topics related to self-care and emotion regulation), Stressor (topics identifying precipitating stressors), and Resource/Process (topics related to service utilization and session administration). All values were derived from the Structural Topic Model with K = 12 topics and spectral initialization.*
