# Supplementary material for: Temporal dynamics of teen crisis help-seeking following hurricanes: A structural topic model analysis
Source: PLOS Digit Health. 2026 May 12;5(5):e0001393. doi: 10.1371/journal.pdig.0001393 (PMC13166961; doi:10.1371/journal.pdig.0001393)
Supplement: S3 Table — (DOCX) [file pdig.0001393.s007.docx]

**S3 Table.** Statistically Significant Temporal Effects on Topic Prevalence from the Structural Topic Model

| **Topic** | **Category** | **Recovery Period** | **β** | **SE** | **95% CI** |  |
| --- | --- | --- | --- | --- | --- | --- |
| Grief & Panic | Crisis | 61–90 Days Post | 0.048 | 0.022 | [0.004, 0.092] | ↑ |
| Grief & Panic | Crisis | 3–6 Months Post | 0.058 | 0.019 | [0.021, 0.094] | ↑ |
| Abuse & Safety | Crisis | 9–12 Months Post | 0.039 | 0.016 | [0.008, 0.071] | ↑ |
| Abuse & Safety | Crisis | Beyond 12 Months | 0.030 | 0.013 | [0.005, 0.055] | ↑ |
| Family Conflict | Stressor | 9–12 Months Post | 0.038 | 0.019 | [0.001, 0.075] | ↑ |
| Family Conflict | Stressor | Beyond 12 Months | 0.034 | 0.016 | [0.002, 0.066] | ↑ |
| Relationships | Stressor | Beyond 12 Months | 0.059 | 0.019 | [0.021, 0.097] | ↑ |
| Sleep & Self-Care | Coping | Beyond 12 Months | −0.063 | 0.017 | [−0.095, −0.031] | ↓ |
| LA Crisis Services | Resources | 61–90 Days Post | −0.059 | 0.019 | [−0.097, −0.021] | ↓ |
| LA Crisis Services | Resources | 3–6 Months Post | −0.057 | 0.017 | [−0.090, −0.025] | ↓ |
| LA Crisis Services | Resources | 6–9 Months Post | −0.041 | 0.018 | [−0.076, −0.006] | ↓ |
| LA Crisis Services | Resources | 9–12 Months Post | −0.041 | 0.019 | [−0.078, −0.004] | ↓ |
| LA Crisis Services | Resources | Beyond 12 Months | −0.060 | 0.015 | [−0.090, −0.029] | ↓ |
| Follow-up | Process | Beyond 12 Months | 0.016 | 0.005 | [0.006, 0.027] | ↑ |
| Session Logistics | Process | Beyond 12 Months | 0.045 | 0.013 | [0.019, 0.071] | ↑ |

*Note: Table presents statistically significant (p < .05) covariate effects from the Structural Topic Model (STM) prevalence estimation. Effects represent the change in expected topic proportion relative to the reference category (3–30 Days Post-Hurricane). β = unstandardized regression coefficient from STM; SE = standard error; CI = confidence interval. Positive coefficients (↑) indicate increased topic prevalence relative to the reference; negative coefficients (↓) indicate decreased prevalence. The Suicide Ideation & Self-Harm topic showed consistent positive coefficients across all post-30-day periods (β range: 0.013–0.017) but did not reach statistical significance due to the high variance, despite the clinically meaningful trajectory shown in Table 3. Topics not shown (Anxiety & Coping Skills, Academic Stress, Crisis Hotline Protocol) had no statistically significant temporal effects. N = 2,149 crisis text conversations.*
