# Supplementary material for: Temporal dynamics of teen crisis help-seeking following hurricanes: A structural topic model analysis
Source: PLOS Digit Health. 2026 May 12;5(5):e0001393. doi: 10.1371/journal.pdig.0001393 (PMC13166961; doi:10.1371/journal.pdig.0001393)
Supplement: S4 Table — (DOCX) [file pdig.0001393.s008.docx]

## **S4 Table**. Compound Disaster Effects: Topic Prevalence by Disaster Context

| **Topic** | **Single Disaster (n = 558)** | **Compound Disaster (n = 1,591)** | **Difference (pp)** |
| --- | --- | --- | --- |
| ***Elevated in Compound Disasters*** | | | |
| Grief & Panic | 4.0% | 7.3% | **+3.3** |
| Abuse & Safety Concerns | 3.5% | 5.3% | +1.8 |
| Anxiety & Coping Skills | 9.2% | 10.7% | +1.5 |
| Sleep & Self-Care | 9.5% | 10.9% | +1.4 |
| Louisiana Crisis Services | 2.8% | 4.2% | +1.4 |
| Academic Stress | 6.4% | 7.6% | +1.2 |
| Suicide Ideation & Self-Harm | 7.5% | 8.6% | +1.1 |
| ***Reduced in Compound Disasters*** | | | |
| Family Conflict | 12.0% | 7.6% | **−4.4** |
| Crisis Hotline Protocol | 16.1% | 12.4% | −3.7 |
| Relationships & Dating | 14.9% | 11.8% | −3.1 |
| Session Logistics | 9.8% | 8.9% | −0.9 |
| Follow-up & Reconnection | 4.2% | 4.6% | +0.4 |

*Note: Comparison of mean topic prevalence between single disaster (Hurricane Barry, July 2019; pre-COVID-19 pandemic) and compound disaster (hurricanes occurring during COVID-19 pandemic: Tropical Storm Cristobal, Hurricanes Laura, Delta, Zeta, and Ida; June 2020–August 2021) conditions. Difference calculated as compound minus single disaster prevalence. pp = percentage points. Topics are ordered by the absolute magnitude of difference within direction categories. Positive differences indicate topics that are more prevalent during compound disasters; negative differences indicate topics that are more prevalent during single disasters.*
